# Current understanding of the immune potential of B-cell subsets in malarial pathogenesis

**DOI:** 10.3389/fmicb.2023.1046002

**Published:** 2023-01-26

**Authors:** Meenu Kalkal, Jyoti Das

**Affiliations:** Parasite-Host Biology, National Institute of Malarial Research, Dwarka, New Delhi, India

**Keywords:** malarial, immunity, B cells, regulatory B cells, marginal zone B cells, follicular B cells, *Plasmodium*

## Abstract

In the past several decades, our understanding of how B cells are generated and what function they perform has continued to advance. It is widely accepted that B-cell subsets play a critical role in mediating immune response. Surprisingly, human and murine malarial infections cause major alterations in the composition of B-cell subsets in both the spleen and periphery. Multiple B-cell subsets are well characterized in murine models following primary and secondary infection, although in human malarial infection, these subsets are not well defined. Furthermore, a rare known function of B cells includes the potential role of regulating the activities of other cells in the body as regulatory cells. *Plasmodium* infection strongly alters the frequency of these regulatory B cells indicating the immunoregulatory function of B cells in malarial. It is important to note that these subsets, taken together, form the cellular basis of humoral immune responses, allowing protection against a wide array of *Plasmodium* antigens to be achieved. However, it remains a challenge and an important area of investigation to understand how these B-cell subsets work together to provide protection against *Plasmodium* infection.

## 1. Introduction

Globally, malarial is one of the leading causes of morbidity and mortality, especially in tropical countries, and represents the greatest public health problem. There were an estimated 241 million cases of malarial in 2020 as per the recent World Malarial Report 2021 compared with 251 million estimated cases in 2010 (World Health Organization., 2021). Data highlight that despite several efforts to prevent and control the disease, no significant progress has been achieved around the globe. However, the advancement in the examination of the host immune response against the multiple stages of the *Plasmodium* parasite, along with multiple strains of the parasite, has resulted in the identification and characterization of important vaccine candidates (Kalkal et al., 2022a). It is also important to note that these advances have strengthened the concept of an effective host immune response that can be beneficial toward the development of an effective vaccine, which can be influential for the control, prevention, and eradication of malarial infections. Although efforts have been made worldwide to develop a vaccine against malarial with good efficacy, still the search remains elusive. After tremendous effort, only RTS, S, a subunit pre-erythrocytic stage malarial vaccine, has gone through a phase-III clinical trial, although its efficacy is not quite good in terms of providing protective immunity in children and the duration of protection observed seems very short (Olotu et al., 2016).

The outcome of *Plasmodium* infection relies on an intricate equilibrium of pro- and anti-inflammatory immune responses generated by the host (Cicchese et al., 2018). The development of a robust host immune response is essential for parasite elimination and for providing protection against re-infection. Concurrently, the immune responses need to be tightly regulated to avoid immune-mediated pathology to host tissue (Kumar et al., 2019). Initiation of immune response generated by *Plasmodium* infection is very complicated and involves the interaction of the parasite with host cells at both exoerythrocytic and erythrocytic stages. Malarial parasites have a very complex life cycle, which starts with the injection of sporozoites. These sporozoites then migrate to the liver, where they undergo multiple divisions in hepatocytes and result in the formation of merozoites, which enter into circulation (Kalkal and Das, 2019). During blood circulation, these merozoites invade the RBCs, where they replicate through multiple stages such as ring, trophozoite, and schizonts. At the blood stage of infection of the parasite, several pro- and anti-inflammatory cytokines are produced as a response by the host immune system (Day et al., 1999). The initial release of pro-inflammatory cytokines works against the parasite by promoting the appropriate cell-mediated and humoral immunity. Although a sustained and excess release of pro-inflammatory cytokine may be damaging to the host, at this stage anti-inflammatory response comes into action and plays a role by limiting the pro-inflammatory response. Various immune cells actively participate to generate an effective immune response; B cells are well known for providing a humoral immune response, although B-cell-mediated humoral immunity is quite slow to develop and involves a complex process. Recent evidence indicates that the malarial parasite has the ability to modify B-cell functions such as polyclonal B-cell activation and hypergammaglobulinemia which results in an impaired immune response (Rosenberg, 1978; Donati et al., 2004; Scholzen and Sauerwein, 2013). Moreover, the immunomodulatory ability of B cells through the secretion of cytokines during *Plasmodium* infection is a new field of investigation. IL-10-producing regulatory B cells are the only explored B-cell subset during malarial pathogenesis; therefore, more emphasis needs to be given to the regulatory ability of B cells with other regulatory cytokines such as IL-35 (Egwuagu and Yu, 2015) and TGF-β (Huai et al., 2021).

## 2. Immune cells involved in protection against the malarial parasite

Innate immunity is the first line of defense against any pathogen that results in the synthesis and secretion of a multitude of cytokines and chemokines which ultimately activates the adaptive immune system. Malarial immunity is referred to as resistance to infection brought about by multiple processes that prevent the *Plasmodium* parasite from reproducing or that limit their multiplication. The development of optimal immune defense depends on a critical interaction between the two components of the immune system: innate immunity and acquired immunity. Indeed, the immune response to malarial is considered very complex, and it acts differently against each species of *Plasmodium* parasite. Moreover, the immune response is stage-specific, as it is activated differently at different stages of infection. Activation of immune response is also influenced by the age of the host and the host's own genetic makeup and previous exposure history of infections. Furthermore, immunity to malarial can be either anti-disease immunity which confers protection against clinical disease or anti-infection immunity conferring protection against parasitemia.

An innate immune response is evolutionarily old and the first line of defense that involves the use of pre-existing cells. Natural Killer (NK) and Natural Killer T (NKT) cells are major contributors to innate immunity at the liver stage of infection (Dunst et al., 2017). On the contrary, dendritic cells (DCs) and macrophages are the early responders of the innate immune response against parasites at the blood stage of infection (Chua et al., 2013; Yap et al., 2019). In addition, it is important and essential to note that the development of an effective adaptive immune response depends on the activation of the innate immune system. This part of the immune system is responsible for the elimination of the pathogen at the blood stage of infection in a very specific way. An adaptive immune system is driven by two primary mechanisms of immunity, called humoral (mediated through the generation of antibodies) and cellular (mediated by mostly T and B cells). Cell-mediated immunity has crucial roles in providing protection against malarial but is also capable of causing tissue pathology and contributing to the onset of severe malarial in some cases (Beeson et al., 2008). Different subsets of T helper cells such as Th1, Th2, Tfh, Th17, and Treg cells that mediate immune response against the parasite have been explored worldwide (Perez-Mazliah and Langhorne, 2014). CD8^+^ T cells also play an important role in providing protection against *Plasmodium* infection, particularly at the liver stage or exoerythrocytic stage of the disease (Van Braeckel-Budimir and Harty, 2014; Rénia et al., 2020). In addition, B cells also contribute toward protection through multiple mechanisms such as by acting as APCs, by producing antibodies, and by providing several cytokines. B cells also play an immunomodulatory role through the secretion of regulatory cytokines, and such B-cell subset is termed as regulatory B cells (Bregs). Overall, controlling the pathogenesis of malarial requires a finely calibrated activation and balance of both humoral and cell-mediated immune responses.

## 3. Immunological role of B cells in malarial pathogenesis

**Figure 1:**
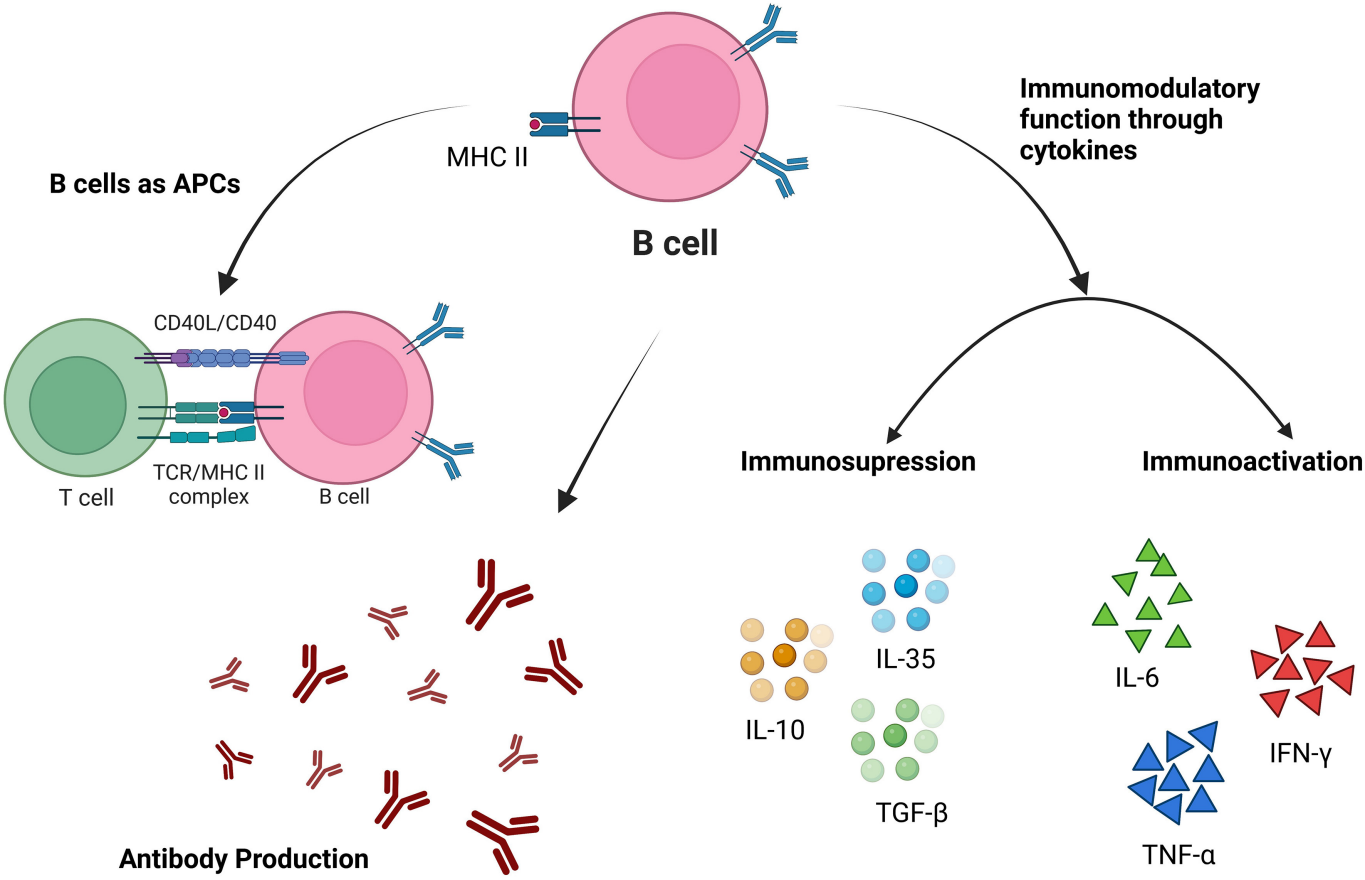
Different immunological functions of B cells in the pathogenesis of malarial.

It has been postulated that humoral response is a key component at the blood stage in malarial, and B lymphocytes/B cells are well-acknowledged for their ability to mediate the humoral immune response by differentiating into antibody-secreting plasma cells. In addition to the production of antibodies, B cells also act as antigen-presenting cells (APCs) to activate T cells and produce various cytokines to participate in the effector immune system (Figure 1).

The development of B-cells takes place in the bone marrow under a complex process that involves the positive selection of functional B cells and apoptosis of autoreactive B cells. B cells are characterized phenotypically by the expression of CD19 which are divided into B-1 and B-2 cells based on their potential to switch immunoglobulin isotype and expression of surface markers (Tarlinton et al., 1995). Two models exist to explain B-cell development to different subsets: the lineage model, which describes that B-cell subsets have distinct progenitors' cells, and the selection model, wherein B-cell antigen receptors (BCRs) decide the fate (Graf et al., 2019). The development of B-1 cells occurs in the fetal liver, which is principally found in the pleural and peritoneal cavities. These B-1 cells are involved in innate immune responses and provide protection by producing antibodies. Furthermore, two different subsets of B-1 cells have been identified as B1a (express CD5) and B1b (no expression of CD5). A striking feature of these B-1 cells is that they express a specific set of Toll-Like receptors (TLRs) and hence can mount a response without the need for triggering by BCR (Prieto and Felippe, 2017). On the other end, B2 cell development takes place in bone marrow as immature B cells migrate into the secondary lymphoid organ, the spleen through peripheral blood. In the spleen, these cells encounter antigens and further differentiate into other B-cell subsets. They are subdivided into immature transitional cells (T1 and T2), follicular B cells (FoB), and marginal zone (MZ) B cells (Wang et al., 2020). Among the all-peripheral subsets of B cells, follicular B cells have the highest level of abundance in the spleen, and they are the progenitors of the subsets of B lymphocytes in development such as plasma B cells, memory B cells, and germinal center B cells (Victora et al., 2012). That is the reason B-2 cells are often used synonymously with classical B cells.

Various emerging studies evidence the capacity of B cells to both activate and suppress immune effector mechanisms as these cells work by both cell-to-cell contact-dependent interactions and through the secretion of various cytokines (Vitale et al., 2010; Cyster and Allen, 2019). The role of B cells in the cellular immune response is now receiving renewed interest owing to studies involving the depletion of B cells (Sacco and Abraham, 2018). Such studies further reveal that B cells not only contribute through antibody production but also provide convincing evidence about their ability to control T-cell-mediated immune response.

Numerous studies based on malarial infection provide a significant body of evidence that suggests antibodies are really important mediators in the development of immunity to malarial. Passive transfer of antibodies isolated from malarial-immune individuals to patients infected with malarial provides protection (McGregor, 1964; Martínez et al., 2009). Furthermore, protective antibodies produced or transferred passively are thought to target multiple antigens expressed by a parasite such as merozoite surface antigens and surface proteins expressed by parasite-infected erythrocytes (Moormann et al., 2019).

In malarial, large numbers of antigens are expressed in each stage of the parasite life cycle including the short asymptomatic liver stage (exoerythrocytic) and the symptomatic blood stage (erythrocytic stage) (Kalkal et al., 2022a). At the blood stage of infection, clinical symptoms of malarial start to appear after a silent liver stage and effective anti-parasitic immunity starts at this stage of infection involving multiple immune cells (Stanisic and Good, 2019). Humoral/antibody or B-cell response represents a critical component of the protective immune response at the blood-stage infection of malarial disease. Antibodies produced by B cells can bind to parasitized erythrocytes that trigger their phagocytosis (opsonization or erythrophagocytosis) by macrophages present in peripheral blood (Osier et al., 2014). Furthermore, antibodies can mark infected erythrocytes for complement system-mediated lysis and prevent the invasion of red blood cells (RBCs) by extracellular merozoites (Biryukov and Stoute, 2014; Castro-Gomes et al., 2014; Boyle et al., 2015). Together, these studies demonstrate that the B-cell antibodies generate an antibody-mediated immune response that works against the *Plasmodium* parasite by reducing the parasite burden but not enough to provide effective control over the disease.

Studies, involving human malarial, have further investigated the potential of antibody-mediated immune response; however, their secretion and functional activity are not much clear. Antibodies are detected in serum within < 2 weeks after infection. Children and adults have been reported to develop short-lived antibodies against malarial infection when exposed to the parasite continuously; however, these antibodies rapidly disappear when there is no parasite re-exposure (Ryg-Cornejo et al., 2016). Therefore, the question arises about the time and their level in serum titers which still remain unsolved (Teo et al., 2016).

**Figure 2:**
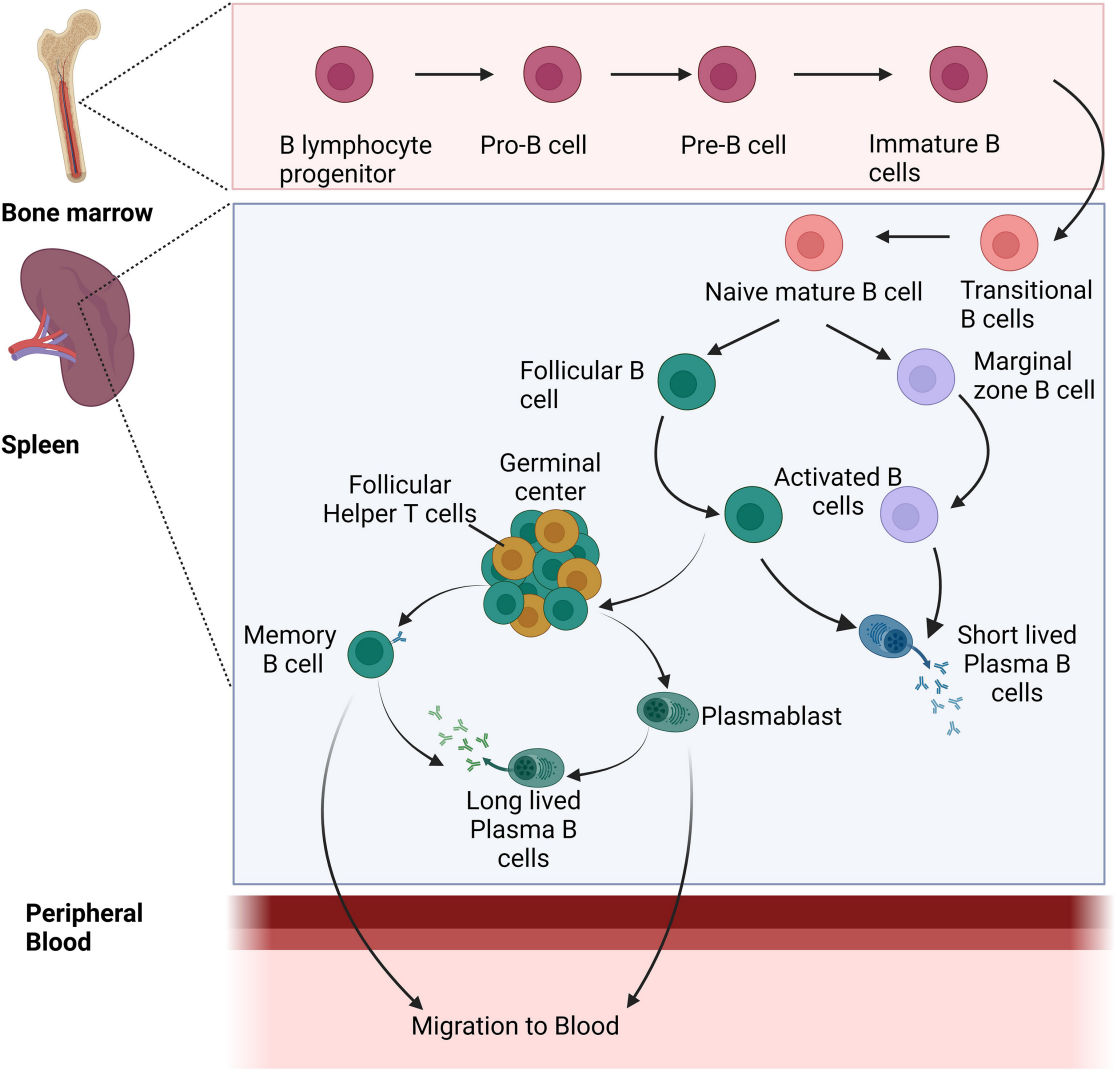
Development and differentiation of naïve B cells into different B-cell populations during *Plasmodium* infection.

At first exposure to the parasite, naive B cells are activated in the peripheral blood upon the interaction of the B-cell receptor (BCR) and multiple antigens expressed by the *Plasmodium* parasite. After activation, these B cells undergo proliferation and differentiation in secondary lymphoid organs which in turn leads to the generation of multiple subsets such as the memory B cells (MBCs), follicular B cells (FoBs), major players of the germinal center (GC) reactions, or marginal zone B cells (MZBs) (Silveira et al., 2018). At first, antibodies are secreted by ASCs or plasmablasts, short-lived cells whose population diminishes after a few days of infection. However, follicular B cells along with the follicular T helper cells (Tfh) and dendritic cells (FDCs) play an important role in the maintenance of germinal centers (GCs). In the germinal center, pathogen-specific B cells are activated which undergo selection that results in the production of antibodies with high affinity and enhanced specificity against the pathogen. These B cells in the germinal center result in long-lived plasma cells and memory B cells with isotype-switching ability and affinity maturation. Long-lived plasma cells help in maintaining the level of *Plasmodium*-specific antibodies, while memory B cells can differentiate into plasmablasts/ASCs quickly upon re-encounter with *Plasmodium* antigen with a greater magnitude as compared to naive B cells (Figure 2).

## 4. Peripheral B-cell subsets involved in malarial pathogenesis

Naive mature B lymphocytes are classically divided based on surface marker expression and function into either B 1 cells or B cells. Transitional B1 cells (B1a and B1b) are mainly present in pleural cavities along with their presence in other organs such as the spleen and the liver. On the contrary, the B2 fraction mainly found in the spleen and lymph nodes further splits into follicular and marginal zone B cells in the periphery. Follicular B cells are the major B lymphocytes in mice, forming germinal centers with follicular T helper cells that trigger highly precise responses to antigens of a protein nature (Nutt and Tarlinton, 2011). While this function is important for the host's survival, the initial response mediated by follicular B-cell responses takes about a week or less before full establishment (Jacob et al., 1991). As an immune response comes into action, there is always a lag and log phase of activation and protection. At the lag phase or initial phase of the immune response, the innate immune system is considered crucial for providing the protection to host, although MZ and B1 B cells are well-established arms of the adaptive immune system. Furthermore, the acquisition of B-cell receptor (BCR) post-recombination of immunoglobulin (Ig) genes along valuation of their involvement in immunological functions reveals that these cells have innate immunity features. Functional analysis of MZ and B1 B cells further discloses that they have the ability to produce plasma cells more quickly than follicular B cells (Martin et al., 2001). Although it is important to note that MZ and B1 B cells are quick responders to non-protein antigens, such as carbohydrate molecules or conserved glycolipids expressed on the surface of most pathogenic organisms (Kearney, 2005), these B cells having physiological similarities to innate cells can identify the number of microbial pathogens and secrete antibodies against them. Notably, this feature could be the major attribute toward bridging the gap between early innate and late adaptive immunity.

Different peripheral B-cell subsets have been described in response to a variety of infections, although very limited literature is available describing the role of these B-cell subsets in malarial disease. In the following section, we have described immature B1 B cells and transitional B cells including T1 and T2 along with mature B-cell subsets such as marginal zone B cells and follicular B cells.

### 4.1. Transitional (T1) and transitional (T2) B cells

These cells are the cells that enter the spleen after they develop from B-cell progenitors. The T1 stage occurs from its migration from the bone marrow to its entry into the spleen, and the T2 stage occurs within the spleen where they developed into mature B cells (Petro et al., 2002). A marked increase in transitional B-cell population along with atypical memory B cells has been observed in acute infection of malarial (Sullivan et al., 2016). On the contrary, another study involving infection *Pcc* AS in the C57BL/6 mice model reported disruption of B lymphopoiesis in the bone marrow. Further this study reported an increase in the apoptosis of transitional T2 and marginal zone (MZ) B cells during infection of malarial (Bockstal et al., 2011). Therefore, it is difficult to explain the population of T1 and T2 cells and their associated function in the context of malarial disease with only a countable number of studies with limited knowledge.

### 4.2. Follicular B cells

Follicular B cells residein secondary lymphoid organs such as the lymph nodes and the spleen. In the context of a thymus-dependent (TD) or T-cell-dependent immune response (TD), FoB cells reside close to T-cell zones in the follicles of secondary lymphoid organs, where at the interface between these two sites these two populations interact (Pillai and Cariappa, 2009). Antigen receptor activation of follicular B cells, along with the assistance of T cell, results in the proliferation of B cells and the development of plasmablast cells. Overall, this results in the formation of the germinal center, the site where B cells undergo somatic hypermutation and isotype switching (Pieper et al., 2013; Figueiredo et al., 2017). These mechanisms produce plasma B cells that have a long life and increased affinity to encounter the antigen in a more specific manner. As a result of this process, memory B cells are also generated for conferring protection against any subsequent exposure to the same parasite in the near future. Using murine malarial models, several studies have investigated malarial parasite-induced alterations or perturbations in the spleen during *Plasmodium* infection which in turn affects the development of B-cell-mediated response (Castillo-Méndez et al., 2007; Bockstal et al., 2011; Pérez-Mazliah et al., 2017). The majority of currently available information is based on the non-lethal *Plasmodium chabaudi* infection model which states that there are alterations in the mice of non-lethal infected mice, and an adequate number of antibodies were found with their ability to act against *Plasmodium* antigens and thus control parasitemia (Achtman et al., 2003; Ndungu et al., 2009). The murine model of non-lethal *Plasmodium chabaudi chabaudi* parasite infection explains that germinal center and marginal zone B cells expand rapidly upon re-infection indicating the generation of an efficient immune response (Stephens et al., 2009). More evidence in the same murine model explained that follicular B cells are maintained in the spleen following infection with acute *Plasmodium chabaudi* AS infection indicating their essential role in controlling the infection (Bockstal et al., 2011). However, unlike non-lethal *Plasmodium* infection, severe forms of lethal malarial infection such as *Plasmodium berghei* ANKA result in the inhibition of the establishment of germinal centers in the spleen (Ryg-Cornejo et al., 2016). Furthermore, histological analysis of the spleen reveals that there are dramatic changes or perturbations such as dissolution of the marginal zone and reduced formation of germinal centers that ultimately have an effect on the development of long-lived plasma cells and memory B cells (Urban et al., 2005).

### 4.3. Marginal zone B cells

Marginal Zone B cells are located between the red and white pulp of the spleen in the marginal zone (Cerutti et al., 2013). In this anatomical niche of the spleen, arterial blood enters and is filtered by the resident cells such as marginal zone B cells, dendritic cells, and macrophages. Therefore, MZ B cells are particularly stationed in such a way that it comes in direct touch with pathogens present in the blood (Zouali and Richard, 2011). Interestingly, various activation markers such as CD80, CD86, and MHC class II are constitutively expressed by marginal zone B cells. Recent studies explain that the MZ B cells have more ability to present antigens to the T cells in a very effective manner than the follicular B cells, thus inducing a stronger response (Attanavanich and Kearney, 2004). It has long been believed that MZ B cells are static or sessile and true that they cannot leave the spleen. However, recent evidence has revealed that MZ B cells are very migratory in nature and they keep on migrating from the marginal sinus area every few hours toward the B-cell follicles (Cinamon et al., 2004, 2008). Even though they make up only a smaller percentage (5%) of the splenic B-cell pool, these MZ B cells along with B1 cells are predominant at an early stage of the immune response T-cell-independent antigens (Martin et al., 2001). MZ B cells are also highly responsive to stimulation of toll-like receptors (TLRs), and upon activation through TLR and PAMP interactions, they differentiate quickly into short-lived plasma cells that secrete IgM (Oliver et al., 1997). As a result of these observations, it has been concluded that these cells bridge the gap between early non-specific humoral response and late affinity matured responses dominated by FoB cells (Lopes-Carvalho and Kearney, 2004).

Phenotypically, FoB and MZ B cells are known to differentially express surface IgM, the complement receptor CD21/35, and low-affinity IgE receptor CD23 that help in phenotypic characterization of these cells through flow cytometric-based strategies under various disease conditions (Baumgarth, 2016). A recent study has emphasized the IgM-expressing B cells in a T-cell-independent manner that results in rapid and avid immune response upon rechallenge to an infection that may be attributed to marginal zone B cells (Krishnamurty et al., 2016). At the same time, there is existing evidence that states that malarial exposure is associated with a decrease in the population of MZ-like B cells and an increase in the atypical MBC population in the spleen (Asito et al., 2008). However, their role during severe infection of malarial is unclear, and a recent study has explained the reduction in the frequency of TACI^+^ MZ-like B cells and a higher expression of CD95 with increasing malarial exposure (Ubillos et al., 2017). TACI deficiency is generally known for an impaired antibody response to polysaccharide antigens, which may explain the increased vulnerability to infection with encapsulated organisms associated with malarial (Tsuji et al., 2011).

### 4.4. B1 B cells

B1 B cells are a subset of B cells dominantly present in the extra-peritoneal and pleural cavities of an organism. These cells contribute to T-cell-independent immune responses and can be further classified into B1a and B1b cells based on the surface expression of the phenotypic marker CD5 (Cunningham et al., 2014). B cells that express CD5 are B1a cells and those that do not express CD5 are B1b B cells. B1a B cells are known to secrete IgM antibodies as the first line of defense against any infection (Haas et al., 2005). On the contrary, B1b cells can also be induced to secrete antibodies to T-cell independent antigens that help in the clearance of a pathogen and help in generating long-term immunity (Alugupalli et al., 2004). The development of these cells occurs in the one marrow, and two models exist describing the development of these cells, lineage, and selection model (Dorshkind and Montecino-Rodriguez, 2007). The basis of the lineage model is fetal liver progenitor cells which can generate B1 cells and B1 cell compartments with a self-renewal capacity of B1 cells (Montecino-Rodriguez and Dorshkind, 2012). On the contrary, the selection model as described earlier suggested that there is a common progenitor precursor cell for both the B1 and B2 cell lineages (Graf et al., 2019). Although B1 cells have not been studied in the context of *Plasmodium* infection, only study involving murine model with *Plasmodium yoelii* 17XNL infection characterized the splenic B1 B-cell plasmablasts during infection with their ability to express granulocyte-macrophage colony-stimulating factor (GM-CSF) and interleukin-3 (IL-3) cytokines during malarial pathogenesis (Chin et al., 2019). GM-CSF is well known for its function in promoting the differentiation of granulocytes such as eosinophils, neutrophils, and basophils (Bhattacharya et al., 2015). Similarly, IL-3 promotes the functional activation of neutrophils and macrophages along with the differentiation of hematopoietic progenitor cells to myeloid progenitor cells (Dougan et al., 2019). Therefore, considering the importance of B1 B cells and their role in protection as part of the innate immune response at an early stage of infection, further exportation of these cells is required in terms of lethal and non-lethal malarial.

## 5. An important novel subset of B cells with immunoregulatory ability in malarial: Bregs (regulatory B cells)

Mizoguchi et al. for the first time demonstrated the suppressive role of B cells during chronic intestinal inflammatory conditions and named them as regulatory B cells (Bregs) with their investigation in T-cell receptor α mutant (TCRα-/-) mice (Mizoguchi et al., 2002). IL-10-producing B cells can be found in mouse spleen at frequencies of 1%−3% and also in smaller numbers in the blood, the lymph nodes, Peyer's patches, the intestinal tissues, and the central nervous system (Tedder, 2015).

### 5.1. Suppressive function of regulatory B cells

In addition to regulatory T cells (Tregs) which exert regulatory function through IL-10 and TGF-β secretion, the regulatory effect of regulatory B cells is exerted *via* the production of regulatory or anti-inflammatory cytokines such as IL-10, TGF-β, and IL-35 (Shen et al., 2014; Wang et al., 2014). In addition to cytokines, Breg cells mediate immunosuppressive mechanisms through Granzyme B and surface molecules such as CD1d, PD-L1, and Fas-L (Catalán et al., 2021). A wide range of autoimmune diseases, cancers, organ transplantations, and infectious diseases have demonstrated immunoregulatory function mediated by regulatory B cells through the production of IL-10 cytokine (Fillatreau et al., 2002; Clatworthy, 2014). In autoimmune diseases, these regulatory B cells with regulatory function work by suppressing immunopathology (Yang et al., 2013; Shen et al., 2014) and dampening immunity during cancer and tumor (Sarvaria et al., 2017; Michaud et al., 2021; Mirlekar et al., 2022). The function of Bregs has also been shown to control immunopathology in various infections such as Leishmania, Schistosoma, and Salmonella (Clatworthy, 2014; Shen and Fillatreau, 2015; Soares et al., 2017). Since the establishment and discovery of regulatory B cells in 2002, it has been almost two decades. Still, there are gaps in understanding the mechanism of immunosuppression mediated by this subset of B cells, and very limited studies under *in-vivo* conditions have described the therapeutic ability of these cells by controlling inflammation during excessive inflammation. With this understanding, it is very clear to state that regulatory B cells work through immunomodulation under various disease conditions, infections, inflammatory diseases, and autoimmune diseases and can be considered a good therapeutic candidate. However, the importance of IL-10-secreting regulatory B cells in the regulation of immune response and immunopathology in malarial remains elusive. Very limited studies have described the expansion and role of regulatory B cells in immunomodulation during infection of the malarial parasite. Moreover, it appears that these cells can influence susceptibility to disease or resistance to disease in a dynamic manner that is affected by the species, strain, and pathogenicity of *Plasmodium* parasites. Therefore, to better understand how these B cells modulate immunological responses during malarial pathogenesis, further studies are necessary. We believe that if attention is paid to exploring regulatory B cells in the context of malarial, we will be able to gain a deeper understanding of the mechanisms by which B cells mediate immunity in malarial. This enhanced knowledge could prove crucial in the development of therapeutics and vaccines against malarial involving the stimulation of B cells. Certainly, in malarial, few murine studies have shown that Bregs are involved in dampening inflammation and some others have explained their immunosuppressive nature *via* the production of IL-10. In a similar manner to murine Breg cells, the human Breg cells have also been described to have immunoregulatory abilities under a variety of different disease conditions, but their involvement in human malarial has not been investigated yet.

### 5.2. Regulatory B cells and their role in mediating immune response in malarial

Various studies involving the blocking of either IL-10 or the IL-10 receptor have demonstrated the importance of IL-10-producing regulatory cells. Furthermore, it also has been described that not all IL-10-producing B cells may contribute equally to immune suppression.

**Figure 3:**
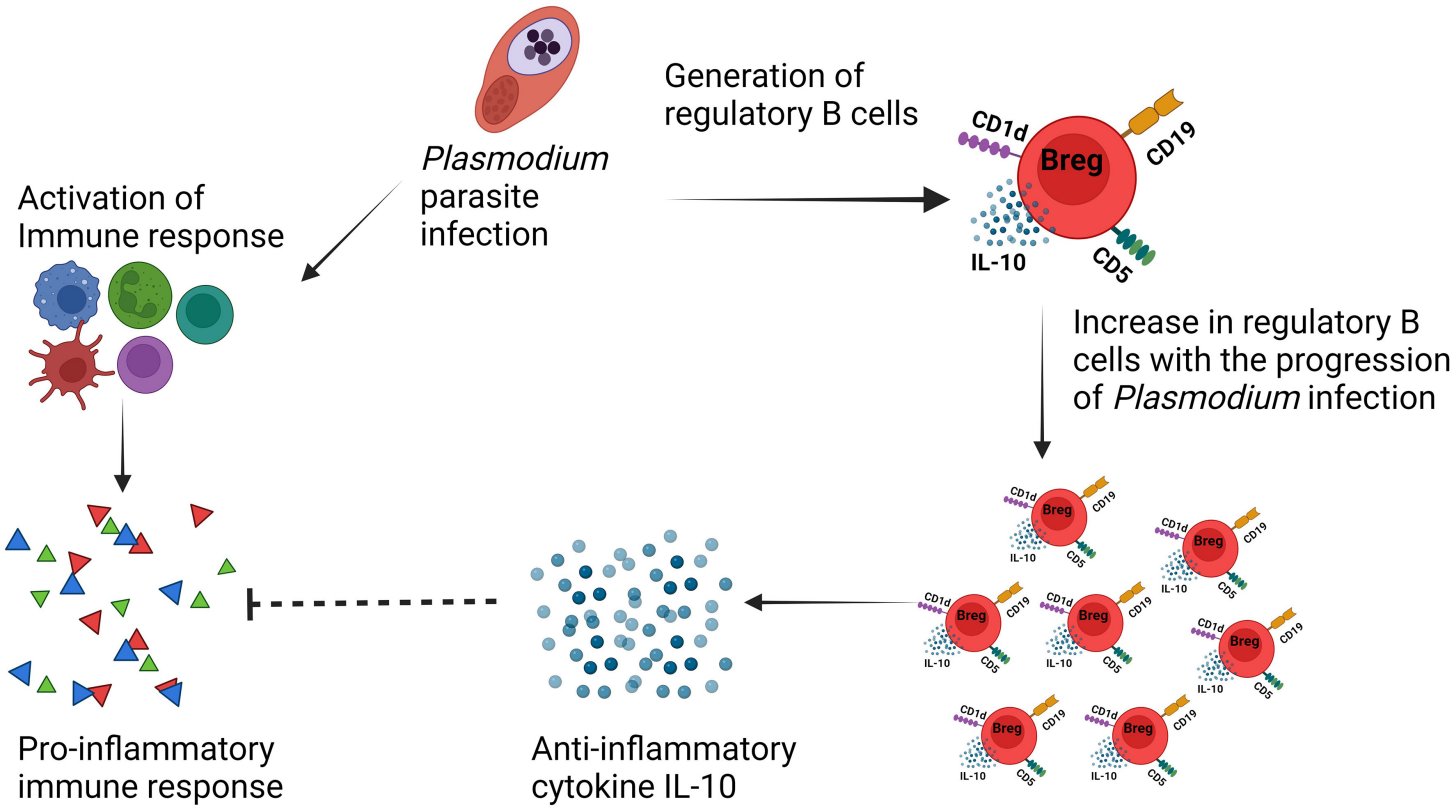
IL-10-producing regulatory B cells mediated immunosuppression during malarial pathogenesis.

Malarial pathology is facilitated by pro-inflammatory cytokines and other mediators that activate and recruit cytotoxic cells to the infection site. A role for regulatory cells and regulatory cytokines has been suggested in multiple pathologic processes through the prevention of the progression of malarial pathology. In uncomplicated malarial, despite the absence of direct investigation involving regulatory B cells in humans, an increase in IL-10-producing B cells in circulation and B-cell activating factor (BAFF) levels in plasma (Nduati et al., 2011) has been observed. In particular, a limited number of studies have explained the relationship between *Plasmodium* infection and IL-10-producing regulatory B cells which are purely based on experimental/murine models of malarial. A very recent study has investigated the dynamics of regulatory B cells during lethal and non-lethal *Plasmodium yoelii* infection in Balb/c mice. It was observed that regulatory B-cell frequency increases in the spleen following infection with each lethal and non-lethal infection. Furthermore, the protective effect of the adoptive transfer of regulatory B cells has been explained in terms of enhanced survival and inhibited the growth of parasites through inhibiting excess inflammation (Kalkal et al., 2022b). Another study explained the phenotype and immunomodulatory function of Bregs during *Plasmodium chabaudi chabaudi* AS infection in two genetically different mice strains namely BALB/c and C57BL/6. It was found that BALB/c mice are more susceptible to infection with strong IL-10 response from CD19^+^ B cells than C57BL/6 mice indicating the role of regulatory B cells toward susceptibility (Han et al., 2018). It was also observed that there is a heterogeneous phenotype associated with Bregs; however, most of the population was CD19^+^CD5^+^CD1d^+^ in both strains of mice. The validation of these regulatory B cells after adoptive transfer in C57BL/6 mice resulted in an increase in the growth of the malarial parasite along with no impact on the survival enhancement. Furthermore, regulatory B cells expressing Tim-1 (T-cell immunoglobulin and mucin domain-containing molecule-1) were also observed during infection, and a significant increase in the frequency of these Tim-1^+^ Breg cells was seen in both strains of mice at day 8 of infection. Breg cells also have been investigated for their role in resolving neuropathology and preventing experimental cerebral malarial (ECM) during *P. berghei ANKA* infection in a murine model by counteracting the excess of inflammatory immune response (Liu et al., 2013). During infection, it was observed that the frequency of regulatory B cells undergoes expansion, and their number increases as the infection progresses. Furthermore, adoptive transfer of these IL-10^+^ B cells (regulatory B cells) in *Plasmodium berghei* ANKA-infected mice resulted in a significant reduction in NK and CD8^+^ T-cell accumulation in the brain and also prevented cerebral hemorrhage, whereas, using the same susceptible model of experimental cerebral malarial C57BL/6, a different study reported that there is need of three cycles of infections followed by treatment for the development of mice resistant to cerebral malarial. Further analysis of cytokines in these resistant mice indicated more anti-inflammatory response through exacerbated IL-10 production at day 7 of infection and suppressed level of IFN-γ from Day 5. The predominant source of IL-10 production found CD19^+^ B cells in the spleen. Adoptive transfer of these CD19^+^ B cells that are negative for CD5 expression which were isolated from resistant mice provided protection from experimental cerebral malarial (ECM) (Liu et al., 2013). Despite the availability of these studies describing that during severe malarial infection regulatory B cells are beneficial as they suppress exacerbated inflammatory immune response responses through IL-10 cytokine (Figure 3), it is still important to investigate the phenotype, their occurrence in secondary lymphoid organs, and their implications during the pathogenesis of malarial toward resistance/susceptibility. Different studies have shown different phenotypes associated with Bregs cells, and their role also seems to be different. However, the strains of the *Plasmodium* parasite and mice used also seem to have an impact on the behavior of the immune system in the context of regulatory B cells.

However, the mechanism of Breg cell-mediated immune suppression in malarial remains poorly understood and ambivalent. Generally, the regulatory mechanism of Bregs cells involves a diverse array of immunomodulatory cytokines of which IL-10 is the most frequently investigated in the pathogenesis of malarial. Although IL-10 is produced by various immune cell types and exhibits several pleiotropic effects, it is important to note that the pro-inflammatory immune response mediated by the release of pro-inflammatory cytokines and other mediators during *Plasmodium* infection is critical for generating protection. At the same time, regulatory cells (Tregs and Bregs) and anti-inflammatory cytokines are suggested to play an important role in preventing excess inflammation effects in the host. Developing a better understanding of the role of regulatory B cells in malarial may contribute to the development of vaccines and immunotherapy, which can aid in the fight against malarial disease.

### 5.3. Development and process of differentiation of regulatory B cells

After the development of progenitor B cells from hematopoietic stem cells, immature B cells enter the spleen. The development of regulatory B cells and the developmental stage at which they acquire their regulatory capacity are still not clear. Two different models have been defined to explain the development of B cells with regulatory ability (Ran et al., 2020). The first model describes that regulatory B cells are generated by a dedicated lineage of B cells, and based on the expression of some genes in the presence of specific transcription factors, the suppressive function of these cells is defined (Rosser Elizabeth and Mauri, 2015). However, such a specific marker for B-cell lineage like FoxP3 has not been identified yet. Another model which is more likely acceptable hypothesizes that B cells differentiate into regulatory B cells in response to any kind of stimulus may be any infectious, parasitic disease, autoimmune disease, or any other disease condition like cancer/tumor. Furthermore, this model is supported by the presence of various heterogenous phenotypes of Breg cells under different stimuli and disease conditions. Various recent research has confirmed under *in vitro* conditions that multiple soluble factors and cytokines induce the generation of distinct Breg cell populations through multiple B-cell pathways involving CD40, BCR, and TLRs. This heterogeneity in phenotype is the main reason behind the classification of regulatory B cells based on their immunosuppressive function in the immune system.

### 5.4. Phenotype of regulatory B cells

Since several B-cell phenotypes having regulatory capacity have been described, there is a hallmark for the exact phenotype of regulatory B cells. This poses a major challenge in understanding the origin, function, and exact phenotype of regulatory B cells. That is why regulatory B cells are characterized based on their function instead of the process of their differentiation. Functionally, regulatory B cells are defined by their immunosuppressive ability through the production of regulatory cytokines such as IL-10, IL-35 (Egwuagu and Yu, 2015), and TGF-β (Huai et al., 2021). Over the period of a few years, progress has been in the characterization of these regulatory B cells during malarial pathogenesis based on their immunosuppressive activity through the release of IL-10.

In mice, a multitude of Breg subsets having functional similarity and even expression of surface markers have been defined in response to various stimuli. However, the most widely investigated and adapted subset of regulatory B cells in mice is B10 cells with phenotype CD19^+^CD1d^hi^CD5^+^ and IL-10 secretion ability. These Breg cells have been largely known to play a role in various animal models including human and murine under various health conditions such as autoimmunity, transplantation, and infections. Even after 20 years of research since their discovery, still there are no known markers that are exclusive to Breg cells. Research is required to search for such markers of Breg just like Tregs which are CD25 positive with the expression of FoxP3. T-cell immunoglobulin and mucin domain-containing protein 1 (Tim-1) is the most broadly identified marker that plays an important role in the induction maintenance of regulatory B cells. It is expressed by the majority of IL-10-producing regulatory B cells (Ding et al., 2011).

Interestingly, the role of Tim-1 appears to be critical for the maintenance of regulatory properties of regulatory B cells. Moreover, B cells that are defective in Tim-1 are more inflammatory rather than regulatory and produce more IL-6, IL-12, and IL-1β than wild-type B cells (Xiao et al., 2015).

During malarial pathogenesis, regulatory B cells have been phenotypically characterized with the limited evidence available. The following table summarizes the phenotypic characteristics of regulatory B cells during malarial pathogenesis. In the following section, a more detailed description of these regulatory B cells and their importance in malarial pathogenesis has been discussed. Table 1 explains the phenotypic characterization of regulatory B cells in malarial pathogenesis.

**Table 1:** Phenotypic characterization of regulatory B cells in malarial.

**Phenotype**	**Mouse model**	***Plasmodium* species**	**References**
CD19^+^CD5^−^IL-10^+^ FoxP3^+^	C57BL/6	*Pb* ANKA	(Bao et al., 2013)
B220^+^IL-10^+^	C57BL/6	*Pb* ANKA	(Liu et al., 2013)
CD19^+^CD5^+^CD1d^hi^	BALB/c and C57BL/6	*Pcc* AS	(Han et al., 2018)
B220^+^CD5^+^CD1d^+^IL-10^+^	BALB/c	*Py* 17XL and *Py* 17XNL	(Kalkal et al., 2022b)

## 6. Conclusion

Significant progress has been made in understanding the immune response initiated by *Plasmodium* parasite infection. Human and animal models reflect the role of various immune cells across the globe in malarial pathogenesis. Nevertheless, a limited number of studies have explained the role of B cells in *Plasmodium* infections. For instance, some preliminary experimental investigations have witnessed changes in the dynamics and expansion of B cells during *P. falciparum* human infection. B cells are considered important for providing humoral immune response although antibodies generated during malarial *Plasmodium* infection are considered very inefficient and short-lived. In addition, atypical memory B cells are generated in response to malarial infection which is an important area of research for the scientific community. Other subsets of B cells along with their mechanism of differentiation into mature B cells need attention for further investigation during malarial that will help us to better understand the malarial vaccine strategies. Studies involving regulatory B cells are not much adequate to understand their role during malarial pathogenesis. Therefore, it is also important to explore the role of regulatory B cells, a novel subset of B cells capable of immunoregulating the immune response during *Plasmodium* infection.

## 7. Clinical perspectives of B cells in malarial

Numerous studies in humans and mice involving B cells very well explain the different immunological functions performed by these cells during *Plasmodium* infection. Multiple B-cell subpopulations actively participate to provide protection against the different stages of the malarial parasite and are responsible for the generation of an effective immune response. B cells in the germinal center further provide more insights toward the generation of antibodies and the formation of long-lived plasma B cells against the malarial parasite. The functional characterization of *Plasmodium* antigen-specific antibodies may provide new opportunities to identify antigenic epitopes that will enable us to identify novel vaccine candidates.

In addition, adoptive transfer studies involving murine models of malarial have explained the immunoregulatory ability of IL-10-producing regulatory B cells. Understanding the immunosuppressive function of Breg cells may open a new way to overcome the excess of inflammatory immune response generated during lethal malarial infection. Overall, it was noticed that B-cell-mediated immune response plays a critical role during malarial pathogenesis. Although several different therapeutic approaches are being explored in the treatment of malarial, it is important to note that these approaches involving B cells are still in the early stages of development. Further research is needed to determine their safety and effectiveness in humans. Moreover, the potential for targeting B cells in the treatment of malarial is an exciting area of research that holds promise for the development of new and improved therapies for this serious and life-threatening disease.

## 8. Future aspects

The information available in the study explained the important B-cell subpopulations in malarial. It was observed that B-cell-mediated immune response has not been characterized very well during *Plasmodium* infection. Therefore, new investigations must be performed to understand the development and activation of B cells along with the establishment of germinal centers. Moreover, the mechanism of immunosuppression of regulatory B cells in the pathogenesis of malarial is also important to the purpose of cell-based immunotherapy. Overall understanding of B-cell subpopulation during malarial infection will be a boost in reducing the mortality associated with the disease, and the novel insights will prove fundamental for effective vaccine design strategies.

## Author contributions

MK and JD conceptualized the topic and content of the manuscript. MK reviewed the literature and wrote the manuscript. JD provided valuable feedback that helped to revise the manuscript. All authors contributed to the article and approved the submitted version.
